# State of the art in total body PET

**DOI:** 10.1186/s40658-020-00290-2

**Published:** 2020-05-25

**Authors:** Stefaan Vandenberghe, Pawel Moskal, Joel S. Karp

**Affiliations:** 1 Department of Electronics and Information Systems, MEDISIP, Ghent University-IBiTech, De Pintelaan 185 block B, Ghent, B-9000 Belgium; 2 Institute of Physics, Jagiellonian University, Krakow, Poland; 3 Department of Radiology, University of Pennsylvania, Philadelphia, USA

**Keywords:** PET, Sensitivity, PET-CT

## Abstract

The idea of a very sensitive positron emission tomography (PET) system covering a large portion of the body of a patient already dates back to the early 1990s. In the period 2000–2010, only some prototypes with long axial field of view (FOV) have been built, which never resulted in systems used for clinical research. One of the reasons was the limitations in the available detector technology, which did not yet have sufficient energy resolution, timing resolution or countrate capabilities for fully exploiting the benefits of a long axial FOV design. PET was also not yet as widespread as it is today: the growth in oncology, which has become the major application of PET, appeared only after the introduction of PET-CT (early 2000).The detector technology used in most clinical PET systems today has a combination of good energy and timing resolution with higher countrate capabilities and has now been used since more than a decade to build time-of-flight (TOF) PET systems with fully 3D acquisitions. Based on this technology, one can construct total body PET systems and the remaining challenges (data handling, fast image reconstruction, detector cooling) are mostly related to engineering. The direct benefits of long axial FOV systems are mostly related to the higher sensitivity. For single organ imaging, the gain is close to the point source sensitivity which increases linearly with the axial length until it is limited by solid angle and attenuation of the body. The gains for single organ (compared to a fully 3D PET 20-cm axial FOV) are limited to a factor 3–4. But for long objects (like body scans), it increases quadratically with scanner length and factors of 10–40 × higher sensitivity are predicted for the long axial FOV scanner. This application of PET has seen a major increase (mostly in oncology) during the last 2 decades and is now the main type of study in a PET centre. As the technology is available and the full body concept also seems to match with existing applications, the old concept of a total body PET scanner is seeing a clear revival. Several research groups are working on this concept and after showing the potential via extensive simulations; construction of these systems has started about 2 years ago. In the first phase, two PET systems with long axial FOV suitable for large animal imaging were constructed to explore the potential in more experimental settings. Recently, the first completed total body PET systems for human use, a 70-cm-long system, called PennPET Explorer, and a 2-m-long system, called uExplorer, have become reality and first clinical studies have been shown. These results illustrate the large potential of this concept with regard to low-dose imaging, faster scanning, whole-body dynamic imaging and follow-up of tracers over longer periods. This large range of possible technical improvements seems to have the potential to change the current clinical routine and to expand the number of clinical applications of molecular imaging. The J-PET prototype is a prototype system with a long axial FOV built from axially arranged plastic scintillator strips.This paper gives an overview of the recent technical developments with regard to PET scanners with a long axial FOV covering at least the majority of the body (so called total body PET systems). After explaining the benefits and challenges of total body PET systems, the different total body PET system designs proposed for large animal and clinical imaging are described in detail. The axial length is one of the major factors determining the total cost of the system, but there are also other options in detector technology, design and processing for reducing the cost these systems. The limitations and advantages of different designs for research and clinical use are discussed taking into account potential applications and the increased cost of these systems.

## Introduction and overview

The successful clinical introduction of novel imaging systems is based on reliable and high performance detector technology but also requires a clear application and a sufficiently large market. Technology is often introduced in an early phase: TOF systems were already introduced in the 1980s [9, 10, 22, 37, 62, 75, 78], with examples published showing Oxygen-15 water-, C-11 acetate- and Rb-82-based studies. These TOF systems were used for human imaging but were not further developed as they did not seem to be competitive with bismuth germanium oxide (BGO)- and NaI-based systems.

There were also some PET prototypes with a long axial field of view (FOV) (> 50 cm) built before introduction in clinical routine [17, 72]. These systems were demonstrators and did not evolve beyond the prototype stage and were not used in clinical routine. Besides the high costs for these systems, there were also important technical challenges like limitations on the detectors, data handling and 3D image reconstruction, which limited the further development of these system. At that time, PET was also not yet widespread for clinical purposes.

Since the early years, PET systems have been improved steadily with regard to sensitivity and resolution by optimising the detectors and geometry. After the integration of PET with CT, time-of-flight technology has been introduced in clinical PET systems. The trend towards fully 3D acquisitions and longer axial FOV started even before the clinical introduction of PET/CT and TOF [27, 63]. Most recent clinical systems have an axial extent of 15–26 cm, work in fully 3D mode and have a timing resolution in the range of 200–500 ps. This range in axial FOV is quite similar to the early days of PET where NaI(Tl) systems from 1990s already had an axial FOV of 25 cm [45]. A next logical step towards better PET systems is to further increase timing resolution although realising further major improvements in timing resolution (below 200 ps) will require new detector concepts and it may take about 10 years before such technology is mature enough for introduction in a clinical PET system. In the limit of 10 ps [33], reconstruction would not even be necessary anymore. There will however still be the need for attenuation and correction for scatter, randoms. Effects from limited spatial resolution and noise would still be present.

Compared to 10–20 years ago, the current detector technology [36, 48] used in most clinical PET systems today does combine good energy, timing resolution and high countrate capabilities. These detectors have now been used since more than a decade to build fully 3D time-of-flight PET systems. Combined with the progress in iterative 3D reconstruction methods and the increase in computing power, all ingredients are available for the construction of total body PET systems. While the gain for organ-specific imaging is limited, factors of 10–40 × higher sensitivity are predicted for multi-organ scans for oncological indications. Most scans performed with a PET system are nowadays body scans (oncology). As the technology is available and the full body concept also seems to match with existing applications, the concept of a total body PET scanner, considered already many years ago, is finally being put into practice. The motivation for research is stronger with new radio tracers becoming rapidly available and for clinical studies due to the high demand and diagnostic benefits.

The aim of this review is to give an overview of the major challenges and most recent developments towards the construction of total body PET systems and explain the potential gains in sensitivity that will enhance current applications or enable new applications. First, we give a summary of the most recent evolutions in PET technology and clinical PET systems which are now the basis for the construction of the first total body PET systems. The concept of total body PET and the recent developments are described in the next part. Afterwards, we will discuss the choice of axial length of these systems (closely related to the total cost) and the different fields where this technology may have an impact. An overview of technical developments to reduce the cost or increase the performance of total body PET is given in the last chapter. The discussion compares this innovation with other recent developments in nuclear medicine and describes different possible scenarios for justifying these systems in research institutes and clinical centres.

### Clinical PET-CT

In the initial days (1980–2000), PET was a useful research tool and the real growth into clinical applications only appeared during the last 20 years. This growth was closely tied to the approval for medicare reimbursement and aligned with the introduction of PET-CT. The major application is oncologic imaging, but other applications include cardiology, neurology and psychiatry. The increased use of PET has been driven by the availability of cyclotrons, but also companies that distribute FDG which made it possible for hospitals to have a PET system without the complexity of an on-site cyclotron. Other factors were the availability of several new PET tracers and more recently generator produced Ga-68-based tracers. Technical improvements of the PET system (delivering improved molecular information) and the integration of PET with CT (co-registered anatomical information) have significantly accelerated PET exams already. These technical improvements with regard to the PET-CT system are summarised in the next section.

#### Improvements with regard to sensitivity, TOF and spatial resolution

During the last 40 years, PET has been improved with regard to sensitivity and spatial resolution [45]. A system with good spatial resolution is not sufficient; it needs to be combined with sufficient sensitivity. This factor is important as the Poisson noise on the detected emission data leads to noise propagation in the final reconstructed image. High sensitivity is therefore important: the final image quality is related to the obtained signal to noise ratio per voxel, which can be increased by a higher number of detected counts. Compared to the first PET systems, the sensitivity of current systems has been increased by 3 major factors:

1. The use of thick detectors (20–30 mm) with higher detection efficiency (first for BGO, but also more recently L(Y)SO)
2. The removal of axial septa: systems have evolved from 2D to 3D with limited acceptance angles and finally to fully 3D systems
3. Increasing the axial length of the system

Besides increasing the number of detected photon pairs, the information content per photon pair has also been improved by introducing time-of-flight measurements in the most recent systems. TOF PET systems [25, 57] do not only register the detectors where both hits of a coincidence are detected, they also measure the time difference of both photons with a precision of a couple of hundred picoseconds. The time difference is used to localise the position of the annihilation along the line-of-response (LOR). This information is then used in image reconstruction via Gaussian-weighted forward and backprojections with the Gaussian weight the same as the spatial distribution of the TOF kernel [70]. This leads to a reduction in noise propagation and an increase in effective sensitivity [50, 58, 59], which is proportional to the ratio of the object size and the TOF resolution.

**Fig. 1:**
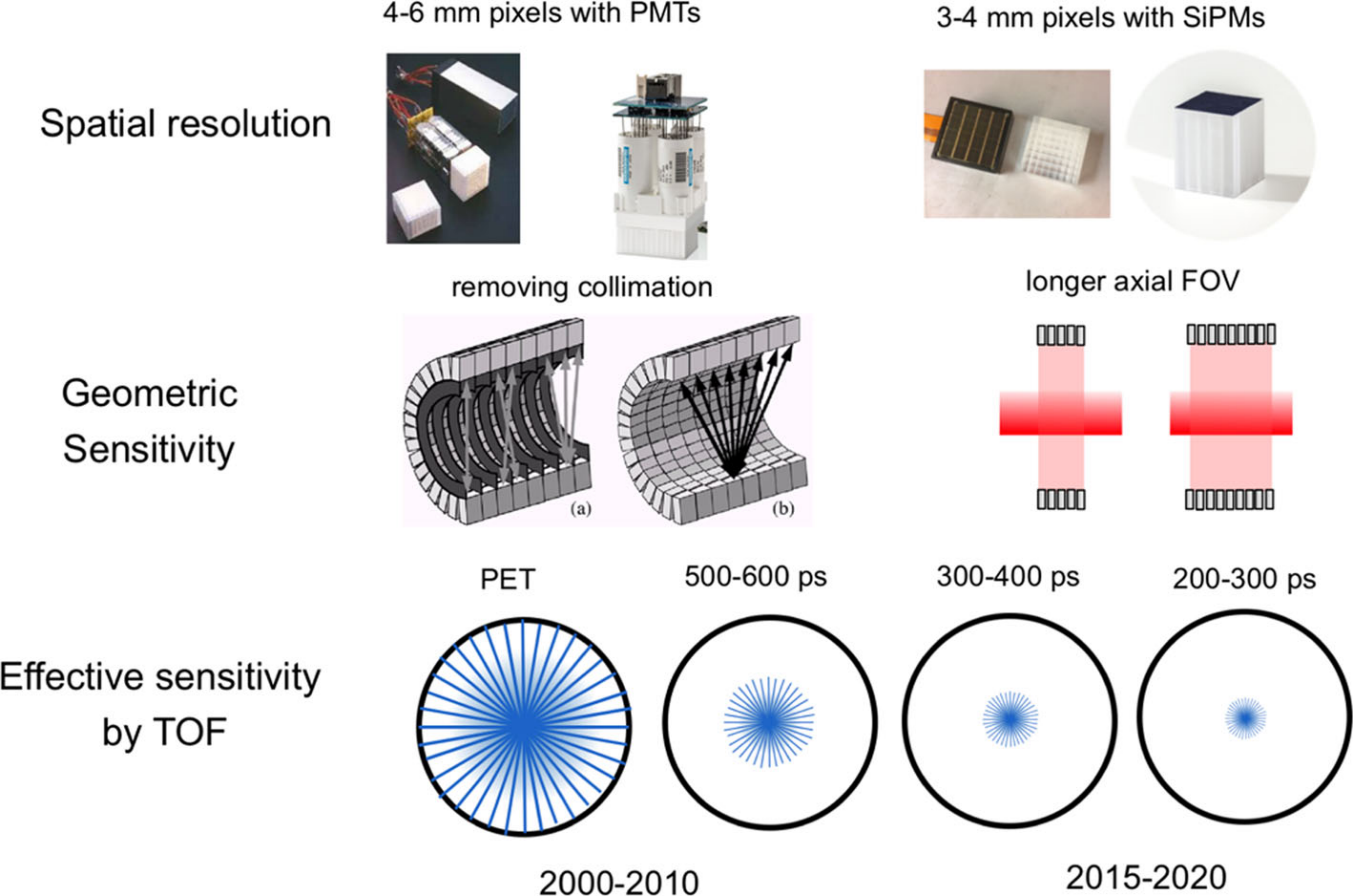
The three major improvements in PET technology during the last three decades

Besides the large improvements in sensitivity, also improvements in spatial resolution have been obtained by using smaller detector pixels and reducing the light spread towards the photodetector. The switch from large conventional photomultiplier tubes to small solid state silicon photomultipliers (SIPMs) [7, 53] has been the latest step in this development. Some of the most recent systems even have one-to-one coupling of scintillator pixels to SiPM pixels. The major improvements in PET system design are shown in Fig. 1.

#### Multimodality imaging with PET-CT

The combination of PET with CT has been very successful and was almost directly introduced into clinical routine. The main reasons are the direct availability and accurate registration of anatomical information with the PET image and the use of the low-dose CT for attenuation correction with a much faster acquisition than traditional transmission scans [6]. The CT image directly provides the required information for attenuation correction [28] and is obtained in less than a minute, while lengthy transmission scans were required for standalone PET systems. Since about 10 years, all commercial whole-body PET systems are integrated PET-CT scanners. The total procedure for one patient in PET currently takes about 20–30 min, including setup: after a quick scout view for selection of axial coverage, a CT of the region of interest (typically head to thigh for a body scan) is acquired in about 1 min and this is followed by the PET study (typically 10–20 min for a whole-body scan). The acquisition length varies depending of the centres preference for lower dose or higher throughput scanning. Acquisition length in some centres may also depend on body mass index (BMI).

Typically, a nuclear medicine department will have a patient throughput of 10–25 patients per day with one or two tracer productions. As the PET acquisition is the slow part in this chain, significantly higher throughput can be obtained by speeding up PET acquisition using systems with higher (effective) sensitivity. During the last few years, there has already been a major improvement by the introduction of scanners with slightly longer axial FOV and better TOF resolution due to the use of SiPM-based detectors. One of the bottlenecks is the time required for putting the patient on and off the bed. This may take 2–5 min as patients selected for PET scanning are often not in good condition.

#### Current whole-body PET design

In the initial years of PET, there were systems based on different geometries, like rotating partial ring systems and flat panel detectors and using different scintillators (BGO, NaI and GSO), but several of these designs and detectors have not been continued in the latest generation of PET systems [45]. Nowadays, nearly all clinical whole-body PET systems have a very similar configuration consisting of an annulus of scintillation detectors surrounding the patient. The bore diameter is typically in the range of 65–85 cm, with the smaller bores being used in clinical PET-MR systems and larger bores in systems adapted for radiation therapy planning. The scintillator used in nearly all new systems is *Lutetium Oxyorthosilicate* (LSO or LYSO) as it has favourable properties for PET like high effective Z and density, combined with good energy resolution, very good light output in a short time and time-of-flight capabilities. Nearly all clinical systems use pixelated L(Y)SO arrays. The pixel size is in the range of 3.2 to 5 mm and its thickness from 15 to 25 mm, resulting in spatial resolution in the range of 3.5–5 mm at the system level. At the system level, good energy resolution of 10–12% is obtained, which limits the amount of scattered coincidences in fully 3D PET. In contrast to 2D PET, the remaining scatter in 3D PET currently still creates unacceptable bias and scatter correction is mandatory.

#### Acquisition mode

**Fig. 2:**
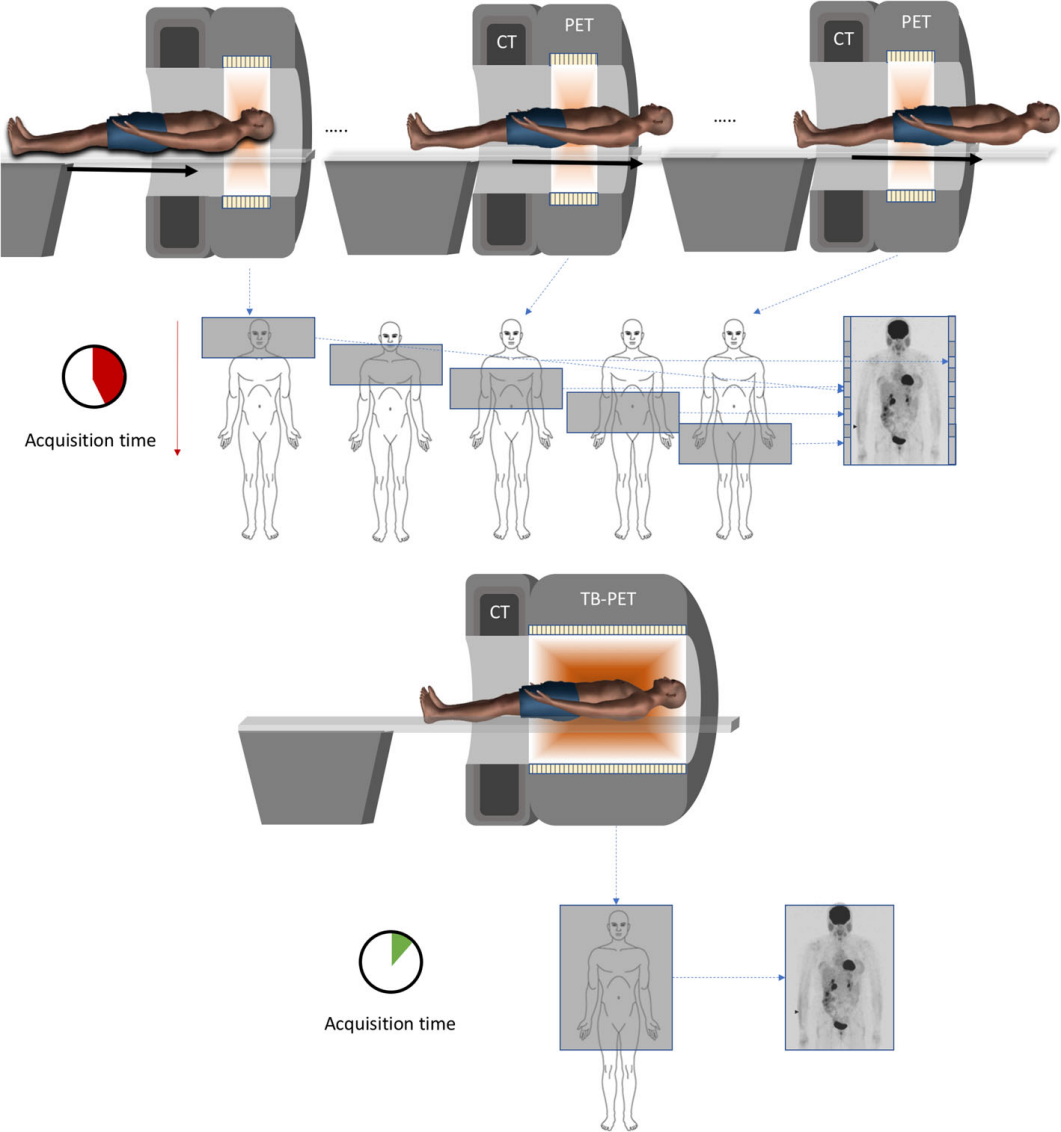
The difference between a current PET-CT (top figure) and a total body PET-CT (bottom figure). Different bed positions to complete a body scan are not required anymore. Inside the total body PET FOV, higher sensitivity is obtained for each point in the FOV by the larger solid angle coverage (indicated by the shading). For the same activity injected in the patient, the total acquisition time can be reduced by a large factor due to the higher sensitivity

The standard axial length of a PET ring (in a state of the art PET-CT system) ranges from 15–26 cm. This is sufficient for acquiring brain or cardiac PET scans in a single axial bed position. Nowadays, the majority of scans performed are in oncology and require scanning a large part of the body with bed translation (typically from head to thigh in about 5 bed positions). These scans are obtained by acquiring the data in step and shoot mode (with some overlap in the axial direction in order to improve uniformity of axial sensitivity) or with continuous bed movement (see Fig. 2). In step and shoot mode, one bed position takes about 1–3 min, the data from the different axial positions (or the continuous movement) are stitched together and body scans are acquired in 10–30 min. An important assumption is that the distribution of the tracer does not change significantly during the acquisition: this is approximately true for FDG studies. Scans are typically acquired at 1 h after injection of this tracer, when the uptake in active regions is sufficiently high and the tracer distribution is approximately at a plateau phase.

## The concept of total body PET

### Limited sensitivity of current PET systems

PET is widely regarded as the most sensitive molecular imaging technique, concentrations down to a pico-molar concentration can be detected, orders of magnitude lower than other modalities. It is however still quite suboptimal from a physics point of view: only a small amount of the emitted radiation from the patient is detected by the imaging system, even the best commercial systems have about 1% sensitivity. A gamma ray pair from an annihilation inside the patient is emitted in random direction; to detect it as two 511-keV photons, several conditions need to be fulfilled:

1. Both photons need to escape the body (not attenuated or scattered [28, 65])
2. Both photons should hit the detector ring (geometrical acceptance)
3. Both photons need to interact in the detectors (detection efficiency)
4. Both signals need to pass the selection criteria (selection efficiency)

The first condition is an intrinsic effect which cannot be avoided. The detector thickness has been optimised taking into account the high scintillation material cost and degradations (like increased depth-of-interaction, DOI) when making the detector too thick. A typical detector thickness for LYSO will be 15–30 mm. Another important improvement of PET detectors has been the combination of uniformity of scintillation performance, improved block detector design and better system calibrations leading to an improvement in energy resolution; most systems will have an energy resolution between 10 and 12%. This allows the use of a smaller energy window which limits the amount of detected scattered photon pairs. This is particularly important for fully 3D PET systems as a poor energy resolution would lead to a very high scatter fraction. The current detectors will typically lead to scatter fractions at the system level in the range of 30–40%. This is according to NEMA NU2-2012, which is representative of measurements with patients of average size.

Two major factors are causing the limited sensitivity in the current PET systems. When the organ of interest is in the FOV, the majority of isotopically emitted gamma pairs will not hit the detector ring due to its limited axial extent. The other factor is that for the specific case of acquisitions of long objects, a large fraction of the object is outside the axial FOV and emissions from those regions can never be detected as true coincidences. With the current axial length of about 20 cm, we only cover about 10–20% of the body’s organs (total body or head to thigh for patients of 160–200 cm) we are interested in.

### Early developments in total body PET

The concept of total body PET (see Fig. 2) is to surround the patient with much more detectors in the axial direction to increase the sensitivity with a large factor.

There are two improvements associated with such a design:

1. First of all, the detection efficiency of photon pairs emitted from a certain point already in the FOV is increased by the larger solid angle (longer axial extent).
2. A much larger fraction of the patient is seen in one bed position, so more FOV is covered in the same time frame.

This idea has already been proposed about 30 years ago (Terry Jones around 1990) and has been the subject of several research studies. Crosetto [18] proposed the three-dimensional complete body screening to maximizing the capture of signals. Besides the slow but steady increase in the axial FOV of the clinical systems, there have already been efforts to built PET scanners with large axial FOV [76]. Hamamatsu has constructed a research scanner with an axial FOV of 68.5 cm [72]. The number of detector rings was 96, and the detector of choice was a 16 × 8 BGO (2.9 mm × 6.3 mm × 20 mm) crystal array readout by flat panel PS-PMT (Hamamatsu R8400-00-M64) [39]. To reduce the number of scattered coincidence events, annular collimators between adjacent rings, called septa, are placed between the block detector rings. The scatter fraction (NEMA NU2-2001) was equal to 31.4%, and the obtained sensitivity was 9.72 cps/kBq. Peak NECR was equal to 113.6 kcps at 10.5 kBq/ml. One main limitation of this system was the energy resolution of the block detector (36% on average). This explains why the system sensitivity was about 40% lower than Monte Carlo predictions.

The early PET systems came with septa because fully 3D data acquisition and reconstruction were not feasible at that time, and then it took some time to verify (using retractable septa) that fully 3D PET was mature enough to replace 2D PET for all applications. In this period, the main bottleneck to fully exploit the total body concept was the performance of the scintillators. Although BGO has a very good stopping power, high detector efficiency alone is not the only factor leading to good image quality. The stopping power however has to be combined with low system dead time, good energy resolution and good timing resolution. LSO, which was introduced in clinical systems in the beginning of the 2000s, is a scintillator with fast timing, good energy resolution and high stopping power. Despite the fact that the cost of the scintillator is clearly higher than BGO, it was quickly introduced in PET, and since 2005, almost all new systems were based on LSO or LYSO with data acquired in fully 3D mode. The LSO scintillator was the basis of a research PET tomograph (Siemens P39-5H) with large axial coverage (53 cm) and composed of five panel detectors mounted in a hexagonal configuration mounted on a rotating gantry [17]. Despite a high sensitivity of 2%, about 2 times higher than current commercial scanners and an acceptable scatter fraction of 40%, the NECR (noise equivalent count rate) did not improve significantly. The relative large detector modules lead to high deadtime losses at high countrates and the system also did not yet have time-of-flight information. For these different reasons, the prototype did not evolve into a clinical system.

After these first prototypes, several simulation studies of long axial FOV systems have been performed [19, 21, 49, 55, 56, 76]. These system simulations have helped to motivate the systems being built today.

### Gain in sensitivity

#### Sensitivity for different sources

Before going more into detail in technical gains by total body PET, it is important to explain the differences in what is understood by sensitivity of a PET system and how it depends on the type of source. We also include equations to describe the sensitivity for generating the figures in this sections.

The sensitivity of a PET scanner is defined as the number of 511-keV photon pairs per unit time detected by the device for each unit of activity present in a source. It is normally expressed in counts per second per microcurie (or megabecquerel) (cps/microCi or cps/kBq). For a preclinical system, the sensitivity is typically given for a point source in the centre of the FOV. Since the major application of clinical PET is nowadays in whole-body imaging, the sensitivity for such a system is determined using a line source with an axial extent of 70 cm. According to NEMA, the sensitivity is measured using a phantom consisting of five nested metal sleeves of known thickness and 700 mm length. Activity is placed in a plastic tube, and this tube is threaded through the inner sleeve. Absolute sensitivity is then obtained by extrapolating to zero thickness. This measurement was defined by NEMA [46] long before there was consideration of actually building systems with axial length beyond 70 cm.

The whole-body sensitivity *S*, defined as the ratio of the registration rate of image forming events (the true coincidences) to the total activity of 511 keV photon pairs created inside the patient, depends on the photons’ attenuation in the body (Att), as well as detection (*ε*_det_) and selection (*ε*_sel_) efficiencies, and may be approximately expressed as:

1 $$ S= \int_{z=0}^{z=AFOV/2} dz \left[ \int_{\theta_{\text{min}}(z)}^{\theta_{\text{max}}(z)} (\epsilon_{\text{det}}(\theta) \cdot {Att}(\theta))^{2} \cdot \sin\theta d\theta \right] \cdot \epsilon_{\text{sel}}^{2} / L_{\text{patient}}   $$

The above formula was derived assuming that the activity is distributed uniformly in the line source with the length of *L*_patient_. Angle *θ* denotes the angle between the direction of gamma photons emitted from the source and the main axis of the tomograph; the term *ε*_det_(*θ*)=1 − *e*^−*μd*/sin(*θ*)^ accounts for the changes of the detection efficiency as a function of the *θ* angle, with *d* denoting the radial thickness of the scintillators and *μ* stands for the linear attenuation coefficient equal to 0.833 cm ^−1^ in case of the LYSO crystal, *Att*(*θ*) indicates fraction of 511-keV photons which does not interact in the imaged object. In the case of the assumed cylindrical phantom with radius of *r*, the term *Att*(*θ*) is approximated by $\phantom {\dot {i}\!}e^{-\mu _{\text {water}} \ r / \text {sin}(\theta)}$ with *μ*_water_≈0.096 cm^−1^. The term sin*θ* d *θ* stands for the angular dependence of differential element of the solid angle, and the angular range *θ*_min_ to *θ*_max_ determines the angular acceptance (solid angle) of the tomograph for the emission from the point *z* along the axis. Selection efficiency *ε*_sel_ (in crystal-based detectors) may be estimated as the photoelectric fraction which for the LYSO crystals is equal to about 0.34. This will underestimate the true efficiency because when the first interaction is a Compton interaction the photon may still be successfully detected.

In the case of the single organ imaging, when the image object is shorter than the AFOV, the integration in formula 1 should be performed over the range from AFOV/2 - *L*_organ_/2 to AFOV/2. The values from attenuation are based on the National Institute of Standards and Technology, NIST database (https://www.nist.gov/pml).

#### Influence of solid angle in long axial FOV systems

**Fig. 3:**
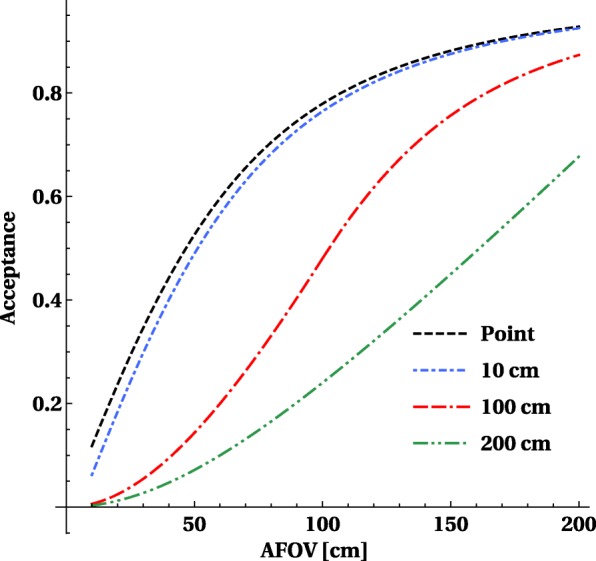
The geometrical acceptance for a point-like source and line sources of 10 cm, 100 cm and 200 cm length in the transverse centre of a PET scanner with a diameter of 80 cm. The *y*-axis shows the fraction of the solid angle

As we make the scanner longer, more LORs emitted from the patient will hit the detector ring, as discussed in detail in [19]. The total solid angle for a point in the centre versus the axial extent of a PET scanner is shown in Fig. 3. At a length of 1 m, already 80% of the solid angle is covered, with 50 cm more than half of the solid angle is covered. So for a single point source (which is an approximate model for single organ), the primary gain is already in the first 50 cm to 1 m and only marginal gains are obtained by extending the PET scanner more in the axial direction.

A line source in the axial direction can be approximated by a set of closely placed point sources, and sensitivity can be obtained by integrating the solid angles for each of the points, see Eq. 1. The fraction of detected over emitted counts for line sources of 1 m length or 2 m length (in the transverse center of the scanner) as a function of axial length is shown in Fig. 3. The choice for a source of 1 m length is motivated by the approximate axial distribution of whole-body organs of interest (e.g., brain to pelvis) for typical human height. It shows that 50% of counts of a 1-m-long source emitted in a 1-m-long scanner are hitting the detector ring, for a 2-m-long source this reduces to 25% of the counts. By increasing the axial length to 150 cm, one can increase the geometric sensitivity above 75% for a 1-m-long source.

#### Influence of detector efficiency and object attenuation in long axial FOV systems on point and volume sensitivity

In a realistic imaging situation, there is also attenuation by the object itself and the detectors are not perfect and have limited detection efficiency.

**Fig. 4:**
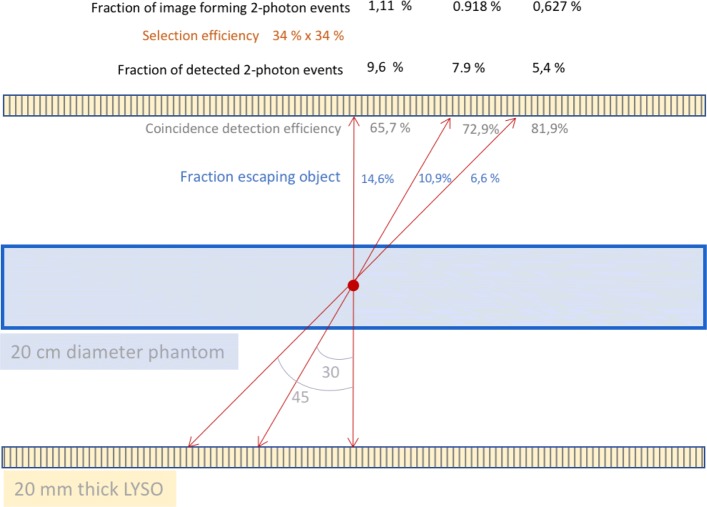
The influence of the oblique angle on attenuation and detection efficiency in a long axial FOV scanner

For large oblique angles, these two effects (one caused by the object and one by the imperfect detection system) counteract each other as illustrated in Fig. 4: the longer the path of an LOR through the patient, the higher the probability of attenuation will be. So if one takes this into account, the gain due to the large solid angle of a total body PET system will be reduced. The effect of attenuation is relatively large for 511 keV as the total path needs to be taken into account (both photons need to escape). A graph of the attenuation versus angle is shown for a phantom diameter of 20 cm.

**Fig. 5:**
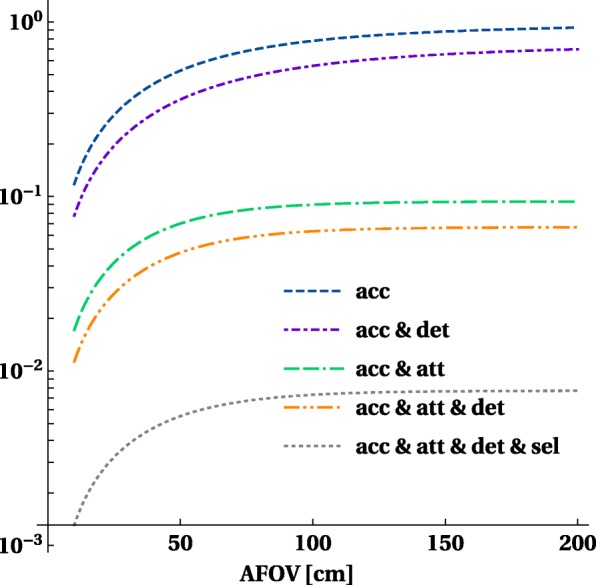
The fraction of detected two-photon events for a central point source taking into account the detector acceptance (Acc), detection with 20-mm-thick LYSO crystals (Acc & Det), attenuation caused by a 20-cm phantom (Acc & Att) as well as (Acc & Att & Det) and selection of event forming events (Acc & Att & Det & Sel). The *y*-axis displays fraction with maximum value equal to 1

On the other hand, LORs at large oblique angles, which escape the body, have a higher probability for detection as they will encounter more detector material due to their obliqueness. In Fig. 5, we illustrate the relative increase for a total body system with perfect detectors (pure solid angle gain) and take the influence of attenuation and detector stopping power into account (assuming 20-mm-thick LYSO).

These curves show that the negative effect of phantom attenuation dominates the positive effect of higher detection efficiency for large oblique incidence. The dashed-double-dot curve in Fig. 5 shows that with a standard PET detector (20-mm-thick LYSO) activity (inside a realistic attenuating object) in the centre of the FOV about 5% of the emitted photons pairs can be detected. This point is reached for a scanner length of about 1 m.

**Fig. 6:**
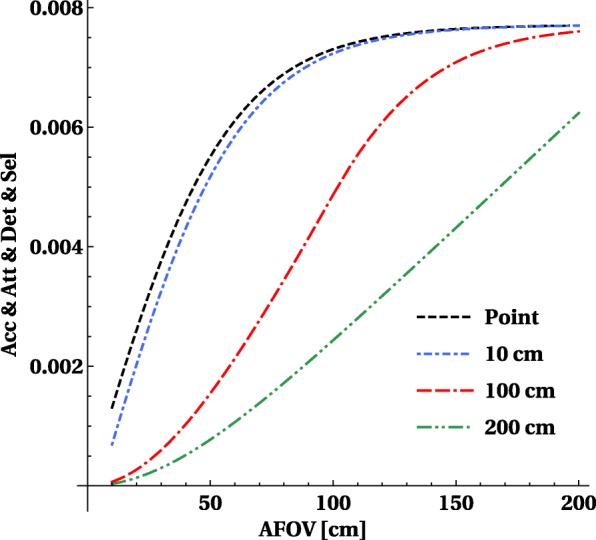
Sensitivity for detection and selection of image forming events for a point-like, 10-cm-long, 100-cm-long and 200-cm-long central line source taking into account the attenuation caused by a 20-cm phantom and the detection efficiency of a 20-mm-thick LYSO detector. The *y*-axis displays fraction with maximum value equal to 1

Using the same methodology as before to calculate the volume sensitivity of the line source, we also take into account the effect of object attenuation and detection efficiency, shown in Fig. 6. This figure is similar to Fig. 3, but shows only the overall sensitivity *S* for different lengths of the line source.

#### How much sensitivity can be gained for different sources?

**Fig. 7:**
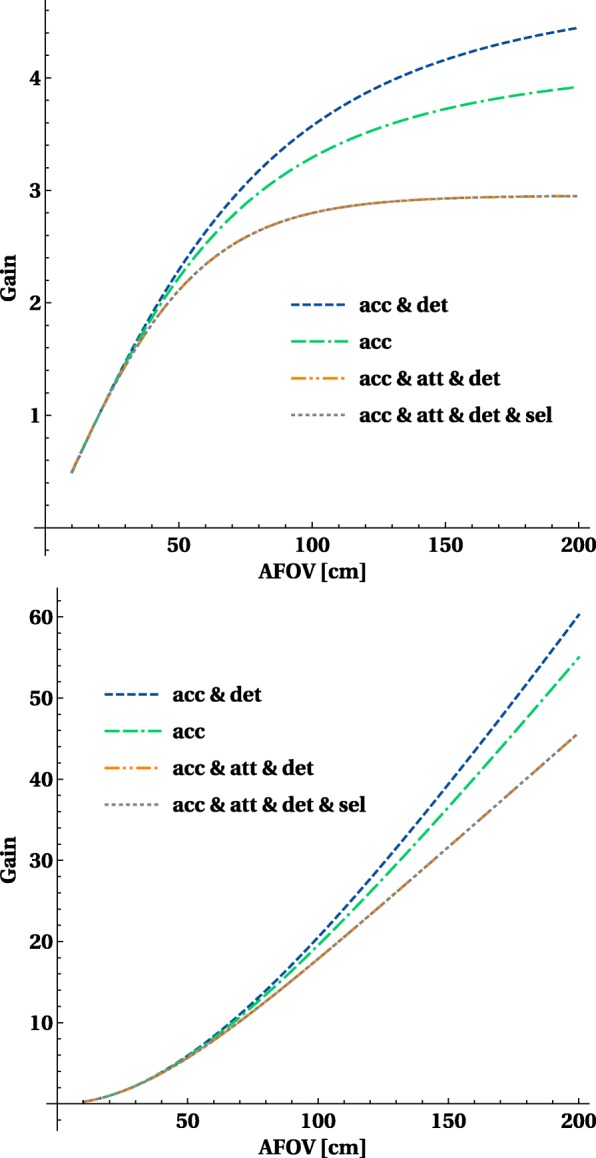
The sensitivity gain versus a 20-cm axial FOV PET system for a central point source, and for a 200-cm-long line source, the curves for pet and det and sel overlap as the gains remain the same. The gain on the *y*-axis is the ratio of sensitivity versus the sensitivity of a 20-cm axial FOV PET system

The ratio versus the sensitivity obtained by a 20-cm axial FOV scanner curves (shown in Fig. 7) show that the gain for a point source is reasonable but limited to a factor 3 and is already reached at an axial length of about 70–80 cm. For extended sources, the gain is much larger and goes up to factors 15 for a 1-m-long scanner and above 40 × for a 2-m-long scanner. Taking into account the detection, efficiency increases the gain, but attenuation has a larger (and negative) effect and reduces the gains.

Point sensitivity is related to human PET scanning where an increased point sensitivity helps to detect and quantify more focal lesions. Volume sensitivity assumes that one is only interested in quantifying the tracer uptake in a bigger volume, like a large portion of the human body. In several cases, we want to have a simultaneous image of the torso and head of a patient; in these cases, we benefit from the large increase in volume sensitivity.

## Total body PET system developments

### Challenges in total body PET with existing PET detectors

Several groups and companies have pixelated PET detectors with the required performance with regard to spatial, TOF and energy resolution for building a total body PET system. When extending systems in the axial direction, the major remaining challenges are mostly related to engineering developments like data handling, cooling and sufficiently fast image reconstruction [83].

#### Detection of coincident events

While the singles rate per detector will be comparable to current systems, there will be a much higher coincidence rate to be handled inside these systems, requiring more advanced coincidence electronics. When making the choice for online storage of all singles events, large datasets will be collected and need to be processed offline to form coincidences [20, 34]. This seems attractive as for example patient-dependant coincidence windows (for oblique LORs larger time differences need to be accepted) can be implemented. It is challenging in this case to keep the combined processing and reconstruction time comparable to acquisition times. However, it has been shown that clinically acceptable processing and reconstruction times are achievable with off the shelf computing power and efficient coding [26].

#### Data size and reconstruction

Iterative methods like OSEM are now the standard reconstruction method for any PET scanner and will very likely remain the preferred method in the near future.

Listmode seems the most natural way to store coincidence events containing information about detector elements and TOF (and eventually energy) information. Listmode becomes favourable when the number of events is smaller than the number of possible sinogram bins. Several new developments (e.g. TOF) lead to a faster growing number of bins than events. The large amount of coincident events per time unit generated by a total body PET system will require the use of extensive processing power to bring reconstruction times at an acceptable level. Another challenge will be the long time storage of the data in this format.

Alternative formats like histoprojections or histoimages use the available information from TOF to position the event into image space before reconstruction. Events are grouped into images or projections [67] according to their polar and transverse angle, and image reconstruction can be reduced to efficient operations like convolution filtering or Fourier transforms with filters [38]. This format does reduce the amount of required data storage per study and can lead to fast reconstruction times independent of the count statistics.

A next generation of reconstruction algorithms based on machine learning [71] may combine the accuracy and fast computation of analytical reconstruction with the higher signal to noise ratio of iterative reconstruction methods. Other authors have implemented a deep learning-based method for accelerating iterative image reconstruction [13].

### Preclinical imaging systems

Several improvements in PET have been introduced first in animal systems as the total component cost of these systems is more within reach of research labs. Besides the early developments in long axial FOV scanners for human imaging, there has been a similar trend in small-animal imaging systems towards long axial FOV. While the first generation systems typically had an axial length in the range of a couple of centimetre [80], the subsequent systems were covering a larger fraction of the animal (typically 8–10 cm). One of the most installed systems, the Inveon small-animal PET scanner, is characterised by a 127-mm axial length but has still a relatively large 161-mm crystal ring diameter. In the last generation systems, the axial FOV (around 12–15 cm) is larger than the transverse diameter and these systems easily cover the complete body of a mouse and a large portion of a rat. The reasons for this early evolution towards total body coverage in small-animal systems are the limited amount of scatter in small objects like mice, which obviates the need for detectors with very good energy resolution. The cost for detector material is also a smaller fraction of the total system cost compared to clinical systems. The newest generation of systems are based on monolithic crystals [30, 54] with different DOI layers enabling a reduction of the detector ring diameter and an extension of the FOV in the axial direction while still improving spatial resolution.

### Explorer project

#### Animal systems

Prior to the first human systems, (large) animal systems have been developed based on clinical technology (Siemens mCT and United Imaging) to demonstrate the potential of new applications and total body imaging capabilities in veterinary imaging. The goal of these systems is to test the technology at a smaller scale and to explore new applications in large animals that may translate to humans. The systems also have a geometry and sensitivity which makes them very suitable for human brain imaging. The first system is called MiniExplorer I [5] and is based on the detectors and electronics from a Siemens mCT (diameter of 87 cm and axial length of 23 cm): the same number of detectors is used but the bore diameter (87 cm) is reduced by a factor two, these detectors are then used to expand the FOV in the axial direction (23 cm) by a factor of two to 45.7 cm, resulting in a system with about 50% solid angle coverage. The system also uses for the first time TOF information for dedicated animal imaging.The system is installed in the California National Primate Research Center at UCDavis. The characteristics of this system are described in Table 1, and first results [82] have been published recently showing 18F-FDG dynamic study of a juvenile rhesus monkey. The high sensitivity enables images of one second frames. A 40-min scan 18 h post injection showed the low-dose capability of total body PET systems.

**Table 1:** Specifications of the developed total Body PET systems for animal (or human brain) use in the Explorer project

System	MiniExplorer I	MiniExplorer II
Scintillator	LYSO	LYSO
Readout	PMT	SiPM
Scintillator size (mm^3^)	4 × 4 × 20	2.76 × 2.76 ×18.1
Total detector elements	32448	NA
Bore/detector diameter	32.0/43.5	NA/52
Axial length (cm)	45.7	48.3
Resolution (FWHM in mm)		
Transaxial	3.0	2.62
Axial	3.0	2.61
Energy res. (%)	NA	11.7
Energy window	425-460	430–...
Scatter fraction (%)		
NU4	16.5 (NU-4)	17.8
NU2-2012	NA	41.9
Sensitivity		
%	5% (NU-2)	12.5% (630 keV)
kcps/MBq		57.16
Coincidence window (ns)	3.6 (46 deg acc. angle)	2.9
TOF resolution (in ps)	609	409
Peak NEC (kcps)		
NU-4 peak monkey	1741 (@158 MBq))	1761.8 kcps@59.4kBq/cc
NU-2 -2012	NA	298.7 kcps@8.4kBq/cc

Several parameters are not yet available as these are systems in development

The second system is called the MiniExplorer II and is based on the detector technology of United Imaging, which is also used for building the human uExplorer. The characterisation of this system has been presented recently at the first total body PET Imaging conference in Ghent, Belgium [1], and the results are shown in the same Table 1. The major differences are the improved TOF resolution (400 ps compared to 600 ps), the reduced pixel size of the detector elements (2.76 mm versus 4 mm) and the slightly increased axial length.

#### Human systems: uExplorer and PennPET explorer

The USA-based EXPLORER [14] program (funded by NIH) program was started in 2015. It has led to the construction of the first total body PET scanner, called uExplorer, by United Imaging Healthcare America, a North American Subsidiary of Shanghai United Imaging Healthcare, and uses SiPM technology from SensL Technologies, Cork, Ireland. The 195-cm-long system has become operational in mid-2018. The detector is composed of 2.76 × 2.76 × 19.1 mm^3^ LYSO pixels. The small pixel size results in a high spatial resolution of 3 mm.The main difference with all other commercial PET scanners is the large solid angle (194.8-cm axial extent), which leads to the highest sensitivity of any PET system. The system is based on an impressive number of crystals (564,480) arranged into 13340 crystal blocks. The number of SiPMs (53,760) is more modest by using a sparse readout method. The system is combined with an 80 detector row CT (in front of the PET scanner). All available specs can be found in Table 2. The system is oriented towards exploring the potential of total body imaging, and the first clinical results (showing the dynamic option, low count studies and fast static scans) have recently been reported in [2].

**Table 2:** Specifications of the developed human total body PET systems in the Explorer project

System	Penn PET Explorer	uExplorer
Scintillator	LYSO	LYSO
Readout	SiPM (digital)	SiPM
Scintillator size (mm^3^)	3.86 × 3.86 × 19	2.76 × 2.76 × 18.1
Total number of detector pixels		564,480
Bore/detector diameter	70/81	68.6/78.6
Axial length (cm)	70/140	194.8
Resolution (FWHM in mm)		
Transaxial	4.0	3.0
Axial	4.0	3.0–3.5
Energy res. (%)	10	11.7
Energy window	440-660	430-645
Scatter fraction (%)		
NU2-2012	32	35.8
Sensitivity		
kcps/MBq NU-2	55	191.5 (@0 cm)
Coincidence window (ns)	5 ns	4.5-6.9 ns (ring difference dependent)
TOF resolution (in ps)	250 ps	505 ps
Peak NEC (kcps)		
NU-2 -2012 (70-cm phantom)	>1200 kcps (for incomplete 70-cm system)	1435 kcps@16.8 kBq/cc
(175-cm phantom)		1718 kcps@8.0 kBq/cc

Parallel to this system, there has also been a major development at the University of Pennsylvania, called the PennPET Explorer [26]. This system is based on the Philips technology used in the Vereos scanner. The detector is an array of 3.86 × 3.86 × 19 mm^3^ LYSO pixels readout by Philips DPC digital SiPM (64 channels per detector). This detector is based on one-to-one coupling between detector pixel and SiPM. The first 3 rings of the system were completed in May 2018, resulting in a system with a 70-cm axial FOV. The current ongoing extension of this system is a further doubling of the axial length of the FOV to 140 cm. First results have been presented at the total body PET imaging conference and are shown in Table 2. The excellent TOF resolution (below 250 ps) was obtained by additional cooling (compared to the Vereos) of the digital SiPMs. This excellent TOF resolution differentiates this system from the uExplorer (400 ps), and the final design of 140 cm axial length will result in a comparable effective sensitivity. Compared to the uExplorer, this system will initially be more oriented to research use, rather than clinical use since it does not yet have FDA510(k) clearance.

### How to use the higher sensitivity?

As current PET imaging is mostly focussed on imaging a relative large part of the body, it is clear that total body PET concept can lead to significantly improved sensitivity compared to the current available systems. This opens different options for its intended use. In general terms, one can use the higher sensitivity in four different ways (or any combination of these).

1. A first option is to keep the acquisition time and administered dose equal and use the higher sensitivity to improve the SNR of images. In several studies, the number of counts is not sufficient to get good image quality, specific cases of these may be the therapeutic isotope Y-90 with very low specific abundance and scanning for example tracers at late time points after several half-lives.
2. A second option is to keep the acquisition time equal and use the order of magnitude in higher sensitivity to reduce the administered dose in vulnerable groups (e.g. paediatric) or for applications where the radiation dose is an important concern. This may also be interesting for imaging tracers with high cost and limited availability (e.g. Zr-89). Also for centers at relative large distance from a cyclotron, this can become an interesting option.
3. A third option is to scan much faster and increase the number of patients per day scanned on a PET system. Especially in areas where the number of PET scanners versus the population is small, there may be a high demand for conventional FDG PET scans.

Besides the higher sensitivity with the potential for faster, low-dose or dynamic scanning, it is also interesting that such a system will give simultaneous information about multiple organs or systems. Several indications may come in the scope of PET imaging. In combination with low-dose imaging, the potential number of indications (also outside oncology) for PET may grow significantly. As this review is focussed on the technical developments, we refer to recent papers on the potential applications of this technology [15].

## Length of a total body PET and potential applications

The cost of a PET system is mostly dominated by the volume of crystals, the area of photodetectors and the required electronics. While it is clear that the full 2-m system is the one with the most flexibility for research, this system is of course also the most expensive solution and may be out of reach for most clinical centers. Another practical consideration of a 2-m system may be a claustrophobic feeling inside the long tunnel of such a system and so display technology inside the bore may be needed to help minimise this effect.

### Axial length

**Fig. 8:**
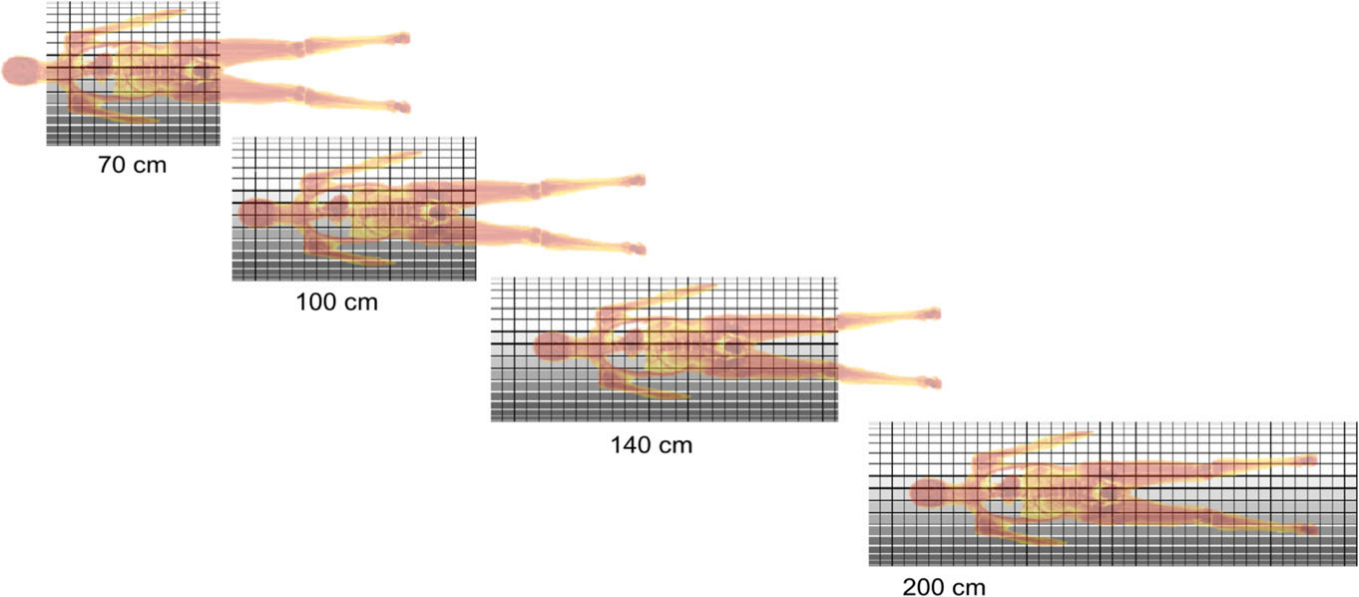
Total body PET systems with an axial length of 70 cm, 100 cm, 140 cm and 200 cm

Several designs have been proposed: all systems have a bore diameter in the range of 70–80 cm but the FOV varies in axial length ranging from 70 cm, 1 m, 1 m 40 and the full 2 m, as shown in Fig. 8. The patient shown has a length of 1 m 70. A 1-m axial length will cover the torso in the majority of population. The sitting height of 95% of American males and 97.5% of the female US population is below 1 m.

**Fig. 9:**
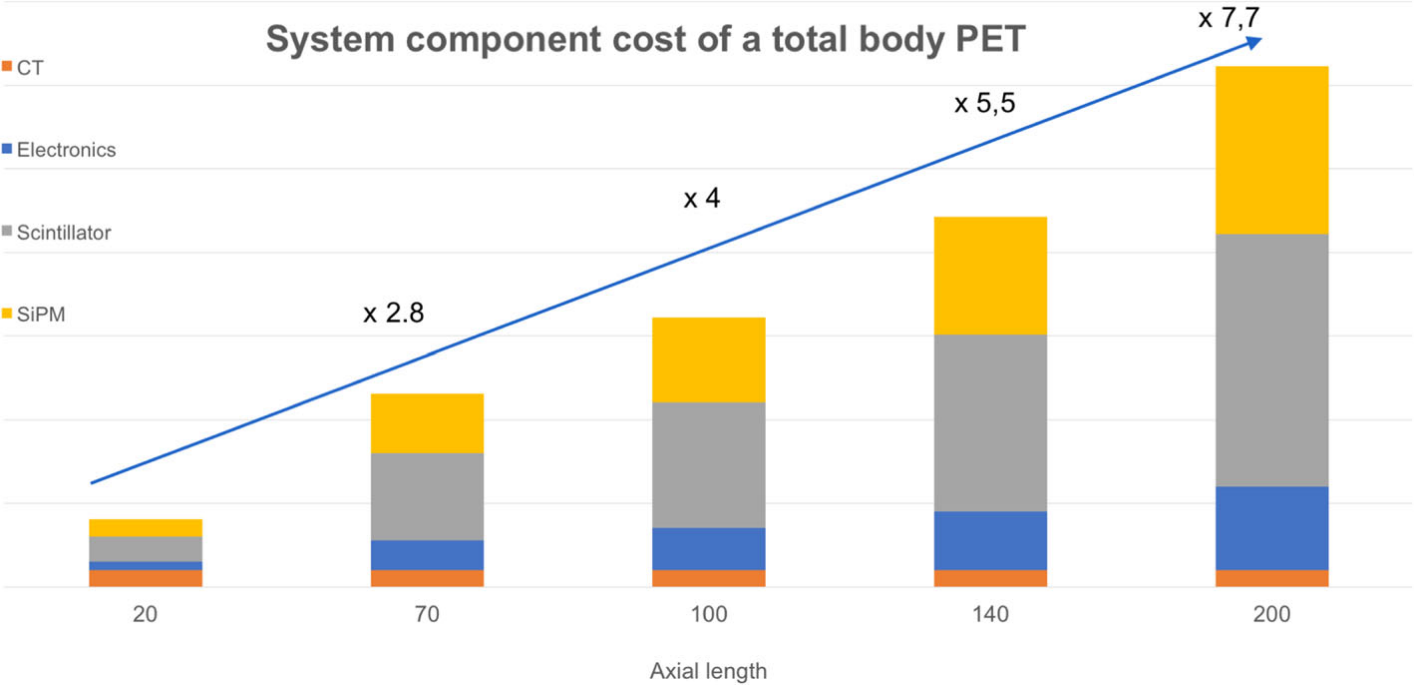
Increase in component costs for a 70-cm, 100-cm, 140-cm and 200-cm system versus a system with 20 cm axial length; the *y*-axis is the system cost in relative units

An estimate of the component cost of a total body PET system versus a system with 20 cm axial length is shown in Fig. 9. The relative cost is based on available prices of CT systems (same for all designs) and quotes from LYSO and SiPMs in large quantities.

**Fig. 10:**
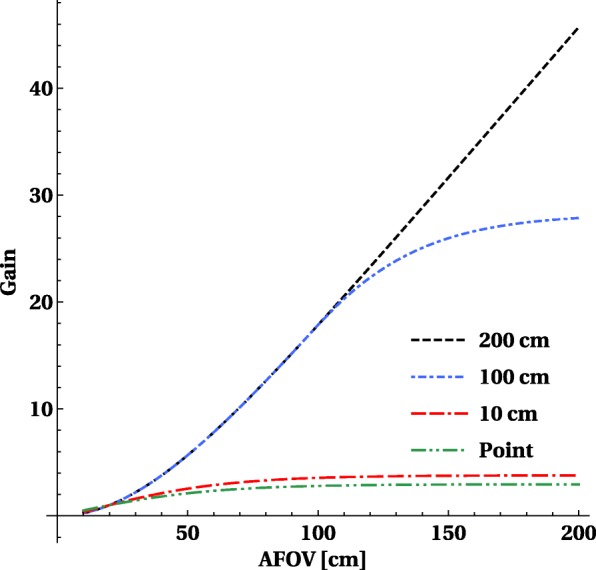
The increase in sensitivity as a function of axial length for a point source, short object 10 cm, a 100-cm-long phantom and a 200-cm–long phantom all filled with activity. The *y*-axis displays the relative gain versus a system with 2-cm axial FOV

The optimal length for introducing total body PET into current clinical routine or research will depend on what the major applications are, and whether the benefits will justify the additional cost for a research institute or a clinical department. Therefore, it is interesting to look at the gain in sensitivity versus a system with 20 cm for different objects, as shown in Fig. 10. We selected a point source representing a single organ, 1-m-long cylinder representing the head + torso and a 2-m-long cylinder for the full body of the patients. For single organ, all systems have a very comparable gain (2.5-3.5 × higher) and the optimum is reached with a 70-cm system. For a 2-m-long object, there is consistent clear gain up to 40 × for a 2-m-long scanner. The slope in sensitivity gain for a 1-m-long object reduces when going beyond 1 m 40.

**Fig. 11:**
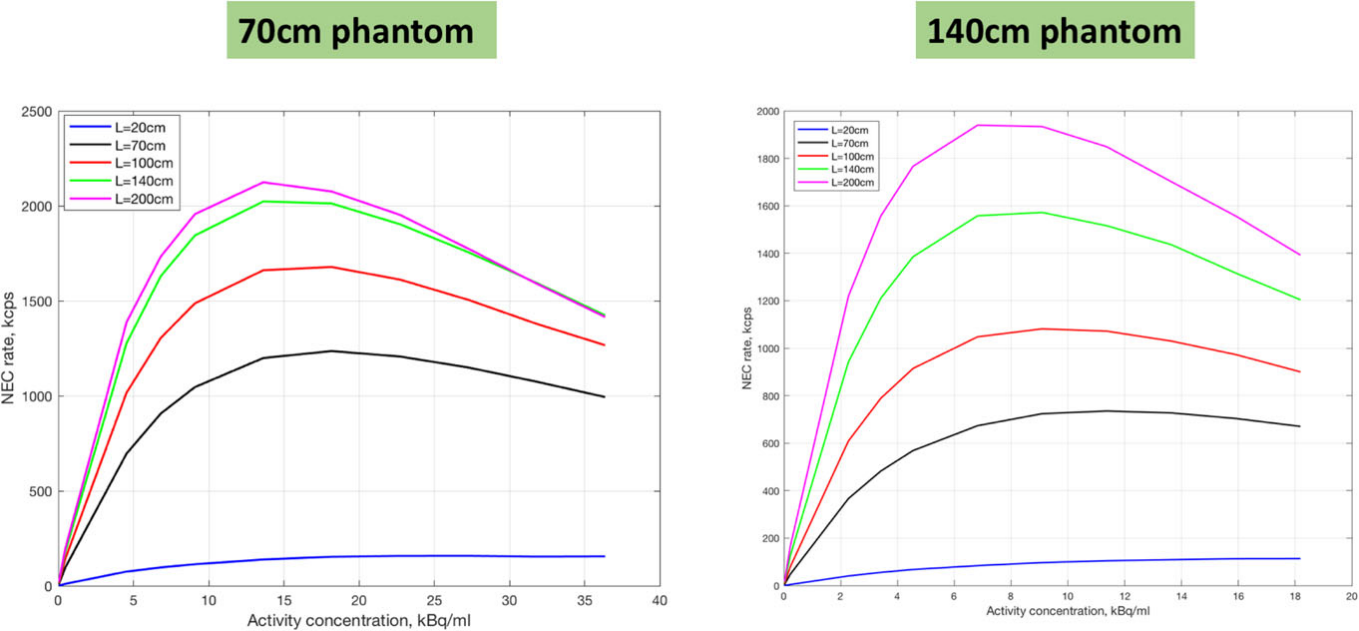
The increase in NEC as a function of axial length for a 70-cm-long and a 140-long phantom for systems of 20, 70, 100, 140 and 200 cm axial length. Simulations are based on a Paralyzable 300-ns dead time per detector block of 5 × 5 cm

Currently, PET scanners are compared based on NECR (noise equivalent count rate). For the same dead time per detector unit (simulations are based on a Paralyzable 300-ns dead time per detector block of 5 × 5 cm), we simulated the NECR for 2 different phantom lengths (70 cm and 140 cm). The resulting curves are shown in Fig. 11. It is clear that the gain for a 2-m-long system compared to a 1-m-long system is moderate for a standard 70 cm. Only for a 140-cm phantom a 2-m-long scanner has a clear increase in peak NECR.

The gains in sensitivity for different designs are summarised in Table 3.

**Table 3:** Gain for different objects and axial length of total body PET systems

Axial length	Gain in single organ sensitivity vs 20 cm axial length	Gain in body sensitivity (1–2-m-long object) vs 20 cm axial length
70 cm	2.5–3.5 ×	9–10 ×
100 cm	2.5–3.5 ×	15–20 ×
140 cm	2.5–3.5 ×	20–30 ×
200 cm	2.5–3.5 ×	30–40 ×

When the primary aim of a total body PET scanner is to use it in current clinical practice for FDG imaging, an axial FOV of 70 cm (9–10 higher sensitivity than current state of the art) up to 1 m is a logical choice. It will allow to reduce the dose, increase the throughput or increase the number of patients per day and is the most economical choice. Designs with a longer axial FOV will be in the first phase research systems exploring the potential of total body PET. They can become interesting for clinical centers for new applications requiring dynamic total body scans or for ultralow-dose scanning.

## Next technical steps

As described before, the major limitation for introducing these systems in clinical routine is the higher cost of such scanners. The major component leading to the high cost is the amount of scintillation material and the second most expensive component is the readout sensor. Some optimisation by distributing the same amount of scintillator over a longer axial FOV [49, 61] is possible, but the total cost of the scintillator material remains high. As the high system cost is a major limiting factor, alternative PET detector technology which could significantly lower costs is of high interest. The high cost of the scintillator material is expected to remain at a high level while only a limited reduction in the costs of the SiPM and readout electronics (due to mass production) can be expected. However, there are some alternative design solutions for reducing the component cost which can have a major impact on the total cost of the systems. One can adapt the geometry, introduce gaps and use deep learning to reduce the effect of low count data.

These solutions are described in the next section, starting with the most evident methods, followed by more fundamental changes in the system.

### Lowering the cost of total body PET

**Fig. 12:**
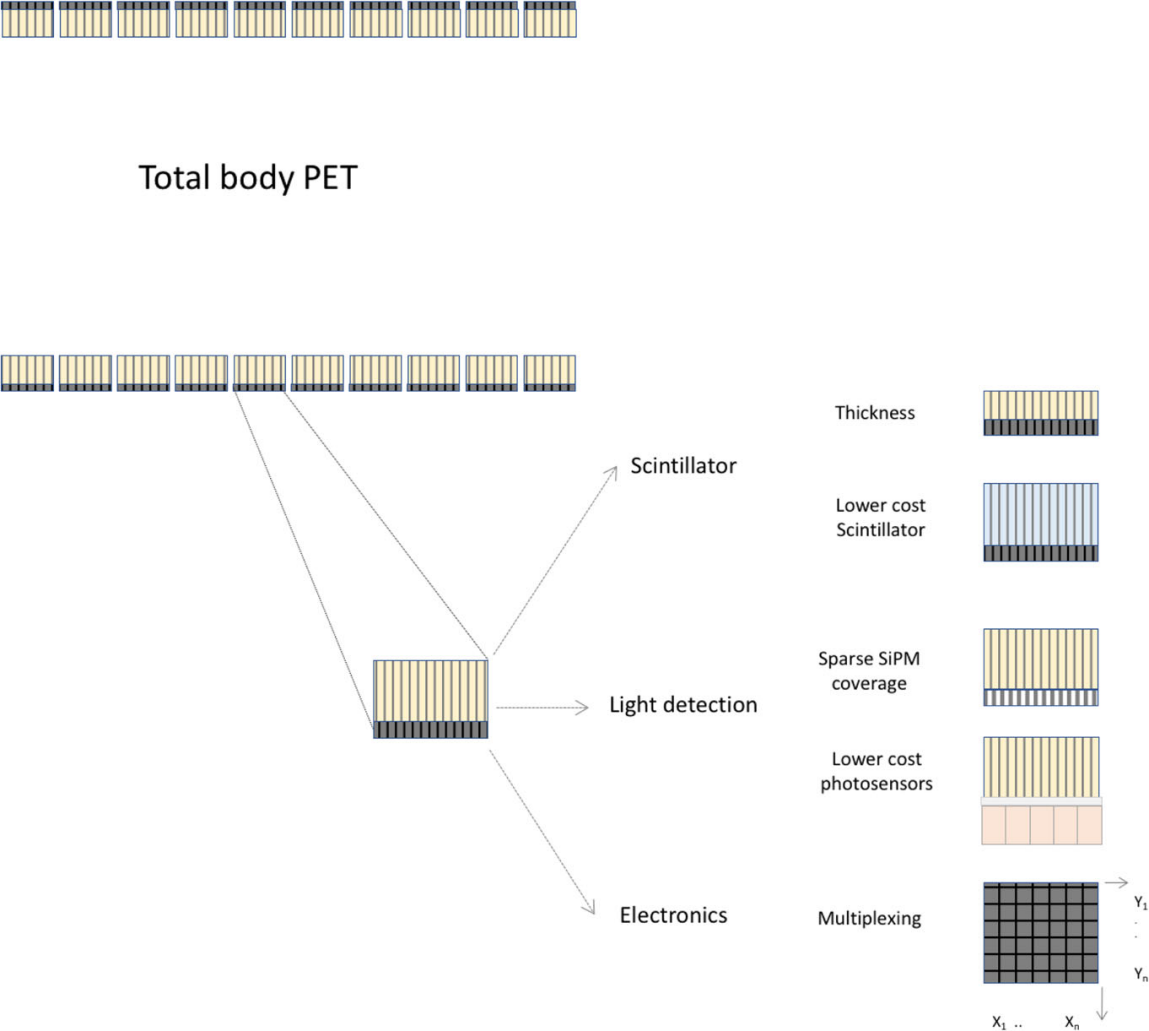
Different options to reduce the cost of the detectors (scintillator, sensor, electronics) in total body PET systems

First, we describe the options to reduce the cost per detector module in total body PET, as illustrated in Fig. 12.

#### Reducing the scintillator thickness

One evident way of reducing the cost of the system is to use scintillators (the most expensive component) with reduced thickness. The major drawback is the fast drop in coincidence sensitivity. A detailed study [61] compared different axial lengths from 20–75 cm but with the same total amount of scintilator volume, so using thinner crystals for longer axial lengths. Keeping the scintillator volume equal to a system with 18 cm axial FOV and 20-mm-thick LYSO, it was found that the optimal system (with LYSO) had a thickness of 10 mm and an axial length of 36 cm.

The loss in coincidence sensitivity may be partially compensated by a better TOF performance for thinner crystals. Several groups have reported TOF resolutions of 100–150 ps for a crystal thickness in the range of 3–10 mm.

### Different detector materials

We have included a table with the properties of the most common scintilllators in PET and added a plastic scintillator (Table 4). Of particular interest are scintillators like BGO and plastic scintillators which are significantly of lower cost.

**Table 4:** Scintillators used in PET

Scintillator	Light output (photons/Mev)	Decay time (ns)	Density (g/cm^3^)	Light attenuation length (cm)
LYSO	32000	41	7.1	20.9
BGO	8500	300	7.13	22.8
GSO	7600	30-60	6.71	22.2
LaBr_3_	65000	15	5.29	16.0
BC-408 (plastic)	11000	2.1	1.023	380

#### BGO

Especially in 3D mode, scanners based on L(Y)SO are performing better than BGO for PET scanners. The main reasons are the higher countrate capability, better energy and timing resolution, explained by the better light yield and shorter scintillation time. This leads to better randoms and scatter rejection. BGO, the scintillator used in the first PET systems, has however a superior attenuation coefficient and higher photoelectric fraction than L(Y)SO:Ce. For the total body PET system design, the advantage of this scintillator is that the cost for the same volume is about 2–3 times lower than L(Y)SO.

While Siemens and Philips are using exclusively L(Y)SO for their PET systems, GE Healthcare still has a line of PET/CT scanners based on BGO detectors [52]. The last generation of clinical BGO-based PET scanners (GE discovery-IQ) is operating in fully 3D mode, has an axial length of 26 cm and has shown an acceptable scatter fraction at the NECR peak of 36.2% by increasing the lower energy treshold to 425 keV. The sensitivity at center of FOV is 22.8 kcps/MBq, which is one of the highest in the field. The detector block is consisting of 6.3 × 6.3 × 30 mm^3^ BGO crystals. The crystals are however relatively large (6.3 × 6.3 ×30 mm^3^) compared to L(Y)SO-based systems which have crystals of around 4 mm transverse and axial dimension and therefore better system spatial resolution. System design studies for a 1-m-long low-cost (pixelated) BGO system have been presented recently [84]. While these systems are based on PMT readout, the combination with improved SiPMs may reduce some of the limitations of BGO. The performance of monolithic BGO blocks readout with SiPMs is quite promising for PET scanners with large volume of scintillators [23].

While initially BGO was considered as a non-TOF capable PET scintillator, this has changed in the last years. The Cherenkov effect leads to an instantaneous photon yield of about 10 photons per 511-keV event. BGO is also a transparent scintillator with a high refractive index of about 2.15. Excellent results have been reported in combination with digital SiPMs by the group in TUDelft [8]. At the single crystal level, excellent TOF full width at half maximum (FWHM) below 400 ps for a crystal thickness of 20 mm has been reported. The full width at tenth maximum (FWTM) values are however relatively high (around 3 ns), which would be expected to have a negative impact on the benefits of TOF-assisted reconstruction. Another important major change has been the combination of BGO with the availability of novel SiPMs working in the NUV region [31]. Using this combination coincidence, resolving time values (FWHM) of about 270 ps from 2 × 3 × 2 mm and about 560 ps from 3 × 3 × 20 mm BGO crystals were measured. When photodetectors with improved response in the near UV/blue response can be developed, better timing resolutions can be expected.

One particular advantage of BGO over L(Y)SO is that for PET studies with very low activity BGO-based scanners do not suffer from intrinsic radiation like Lutetium-based scintillators [21]. This effect has not been studied in detail for total body PET systems, but may introduce some limitation in the case of ultralow-dose imaging due to the relative high amount of scintillator material in these systems.

Right now, BGO seems to be the main competing scintillator for L(Y)SO for a total body PET design as it is also available at low cost and in large quantities. There continues to be research into new scintillators with favourable properties, although these are not yet practical for large-scale production. Another interesting alternative for L(Y)SO may be LuAP [32] as it combines high density of 8.34 g/cm ^3^ with fast response time (17 ns). Also the energy resolution is at least equivalent to LSO. It does however not have a similar cost advantage as BGO. Other interesting scintillators with more light and without intrinsic activity are LaBr_3_ and CeBr_3_. The main disadvantage is the lower density and higher probability for compton interaction (lower photoelectric fraction), although these disadvantages can be minimised by the large geometric sensitivity gain of a long axial FOV system.

**Fig. 13:**
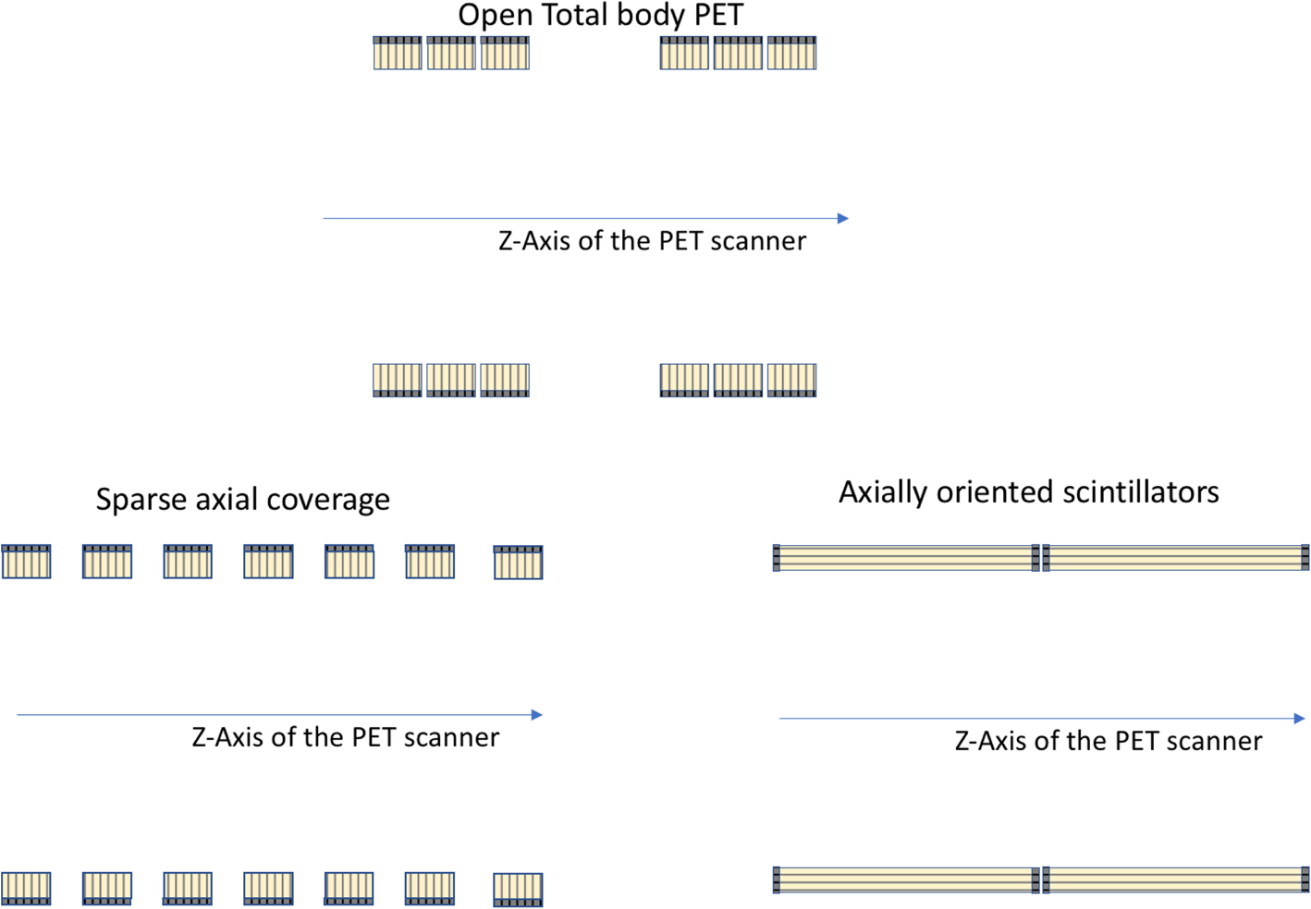
Proposed cost-effective designs for total body PET systems

Besides only changing the detector itself, there are also other options by departing from the conventional multiring approach, as illustrated in Fig. 13.

#### Plastic scintillators

A system built from axially arranged plastic scintillator strips is proposed by the group in Krakow [41, 44]. This technology can be used as an alternative to inorganic scintillators and can lead to cost-effective total body scanner. As the design makes use of much less expensive detector material, the cost for the same volume is about 80 times lower than for L(Y)SO. The mean path is much longer, and to achieve comparable stopping power, a much greater thickness is needed for plastic. In this type of scintillators, nearly all interactions will be due to the Compton scattering, making the discrimination of scatter more challenging than in conventional PET systems.

The reduction of scatter fraction is, however, possible [42] and as it was shown in reference [29], the selection of events with energy loss higher than 200 keV decreases the scatter fraction down to about 35%.

**Fig. 14:**
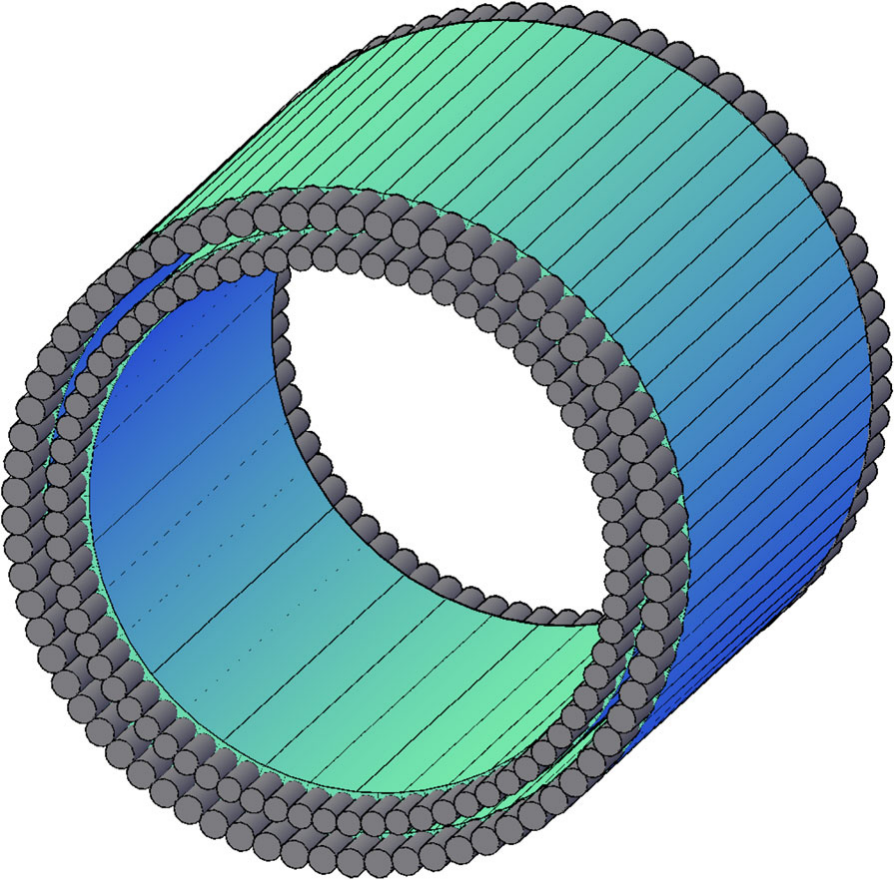
The axial arrangement allows for concentric layers of scintillation material

With plastics strips, the number of electronics channels may be reduced significanty also for the total body PET, because of more than order of magnitude lower light attenuation of plastics compared to crystals [73], and hence application of long strips. In principle, a total body PET may be constructed from two 100-cm-long cylinders or even single 200-cm-long strips since the plastic scintillators’ attenuation length may be as long as 400 cm. So far, a prototype of plastic PET with 50 cm axial length was commissioned [47]. The low density of plastic scintillators (around 1.032 g/cm ^3^) will however require larger amount of detector material. The readout at the edges facilitates also possible application of plastic PET as an insert to MRI or even CT scanners. But if the plastic is made thick enough, it may not be possible to use it as an insert in the bore of a standard MRI or CT. Yet, the axial arrangement enables for application of many concentric detection layers compensating for the low efficiency of plastic scintillators [44] as illustrated in Fig. 14.

**Fig. 15:**
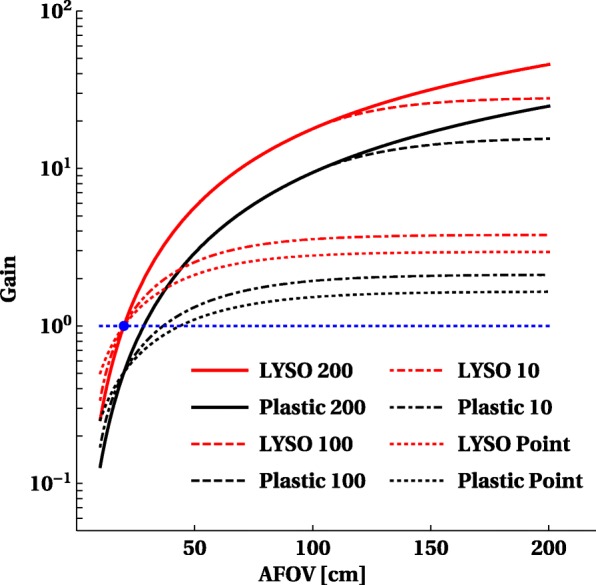
Sensitivity gain, with respect to 20-cm length LYSO PET, as a function of the axial length for LYSO (2 cm thick) and plastic (two 3-cm-thick layers) detectors. Results for a point-like, single organ (10 cm), as well as 100-cm and 200-cm sources are shown

Figure 15 compares the sensitivity gain for the registration of true events with plastic and LYSO detectors. It shows results of calculations (Eq. 1) assuming 2-cm-thick LYSO blocks and 6-cm total thickness of plastic scintillator layers. The figure illustrates that the total body plastic PET with total thickness of 6 cm may increase the sensitivity with respect to the current 20-cm length PET based on LYSO crystals by more than factor of 20, which is two times less compared to the total body PET from LYSO crystals. The mechanical robustness of plastics compared to crystals enables the construction of a light, modular and portable total body PET system. These scintillators are also fast enough to enable TOF measurements [43] to improve noise properties in human body PET imaging. In principle, a TOF resolution below 100 ps is achievable [44, 51].

### Detector configuration

#### Sparse axial coverage of detectors

3D PET contains redundant data, and it is not necessary to fill all axial rings with detectors to obtain tomographic information. Yamaya et al. [79] proposed an ‘open PET’ geometry, consisting of two axially separated detector rings. While the initial ideas were mostly focussed on the benefits for in-beam PET imaging (creating a gap in the axial center), this concept may also be of use for creating total body PET systems with reduced cost. Up to 50% of the detector surface can be gaps at the expense of significant sensitivity loss: by reducing the detectors by a factor of two, the volume sensitivity will drop by a factor of 4. A recent study [81] for a Philips Vereos design showed that removing 50% of detectors in the transverse or the axial direction did not have a major impact on the SUV values. Phantom and human imaging results reported for the initial configuration of the PennPET Explorer [26] were acquired with gaps between the rings corresponding to a data loss of 30% of each ring, thereby demonstrating the potential to trade-off in axial length between sensitivity and total number of detectors.

#### Axially oriented scintillator-based detectors

The detector proposed in the AxPET collaboration departs from the conventional PET detector with radially oriented crystals [3]: long crystals are oriented parallel with the main axis of the scanner and readout on both sides by photosensors. The main advantage of this approach is a reduction in the number of readout channels and parallax free data resulting in a very small degradation of transaxial resolution. This approach may be an interesting option for building scanners with increased axial FOV.

### Reducing the readout complexity

An evident way to reduce the number of channels is to use PMTs instead of SiPMs. As shown in one of the early total body PET designs (using Photomultiplier tubes), the EB-PET by Wong [74], the quadrant sharing approach may be an effective way to reduce the number of readout sensors and channels. While in a conventional PET block design 4 small PMTs are used per detector block, in this design, a larger PMT is used and it is now shared between 4 detector arrays. The EB system is using large 39-mm round PMT, and in this way, the number of PMTs required for a 1-m-long PET is equal to 1768 for the 205,700 crystals. This approach was recently adopted for the uExplorer, but there it is based on much smaller SiPMs. It has the advantage of reducing the number of channels, which is important for expensive devices. On the contrary, there is poor light coverage since these devices are much smaller than PMTs. This has an effect on the timing and energy resolution.

While most clinical systems are still based on PMTs, the cost-benefit ratio of SiPMs has however seen a major improvement and this will have an impact on the final system performance of a PET system. The most recent PET detectors are based on SiPMs, and this readout is also used in the first total body PET systems for human use. The one-to-one coupling used in the Philips Vereos PennPET Explorer is the most evident choice to have the best performance at the detector level. The GE detector does not have 1-to-1 readout and the SiPM does not cover the complete crystal block and so has worse timing. The United Imaging detector block has the least coverage and poorer timing performance. Even with the lower light collection due to incomplete coverage, an excellent energy resolution of 11.7% and good timing resolution of 409 ps is reported.

### Image reconstruction and deep learning

An alternative solution may be the combination of reduced number of detectors (or thinner scintillators) and a further improvement of image quality with methods like regularised reconstruction. Some recent studies have also used deep learning [11] to estimate high count images from lower count studies predicting a possible reduction with a factor of 4 in counts and may be applied to total body PET scanners. Combined with anatomical information, some recent studies even claim a factor of 100 × lower counts [12]. For brain PET-MR imaging data, factors up to 200 × count reduction are claimed with these methods [77]. In another recent paper, the authors have used deep learning to estimate full-dose PET images from 1/10th dose PET images [24].

### Improving the performance of total body PET

**Fig. 16:**
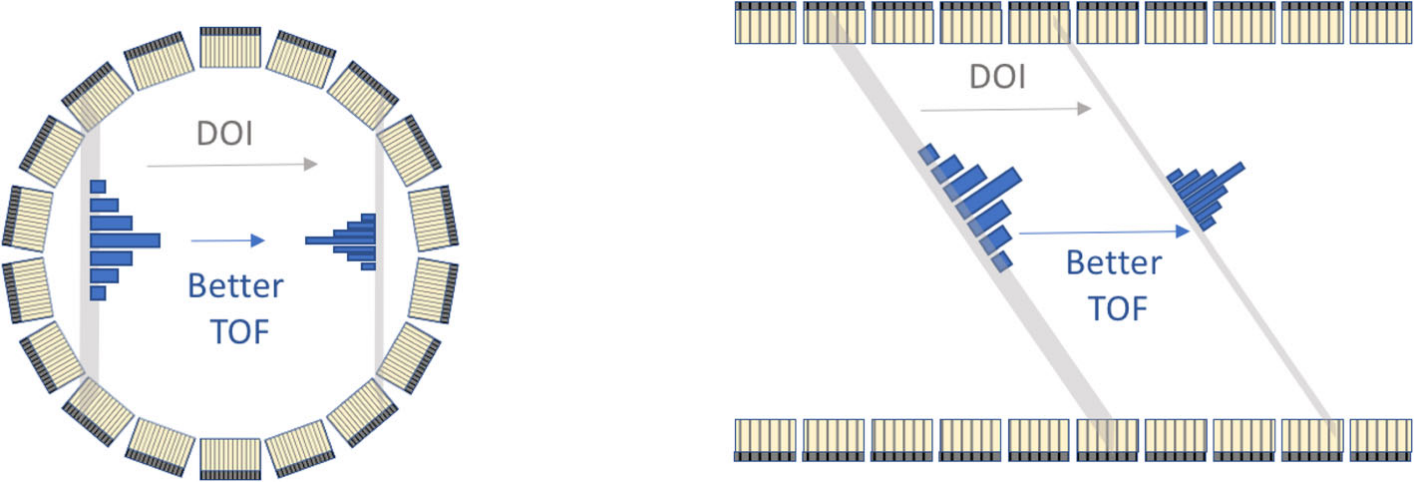
Two possible improvements in future total body PET systems: introduction of DOI and better TOF

Instead of reducing the cost of the detector, there is also the option to further enhance the detectors used for building total body PET. The Explorer design has a full body coverage and maximised the geometric sensitivity but can still be improved with regard to some other parameters. From the technical perspective, there is still room for further improving these systems with d of interaction (DOI). Three possible lines of improvement are the TOF resolution (United Imaging uExplorer has about 500 ps TOF, PennPET Explorer has 250 ps), high spatial resolution and DOI. An illustration of the influence of these improvements in transverse and axial direction is shown in Fig. 16.

#### Improving the effective sensitivity by better TOF

Current PET systems with the best TOF resolution (at the system level) are the PennPET Explorer (250 ps) and the recently introduced Siemens Biograph Vision (214 ps). Several detector groups have shown in a lab setup performance down to 100 ps, often at the expense of detector thickness. The interesting property of TOF is that it increases the effective sensitivity for any object larger than its effective TOF kernel. As 400 ps is already equivalent to a FWHM of 6 cm, gains for any time resolution below 400 ps will be seen in any part of the human body. The axial extension of a PET system is mostly relevant for objects with a length comparable to the length of the scanner. Short axial objects (e.g. Human brain) will therefore have a higher effective sensitivity in a system with 70 cm axial length and 200 ps, than in a system with 140 cm and 400 ps TOF.

Most TOF PET detectors are based on simple signal processing techniques like leading edge discrimination or constant fraction discrimination to estimate the TOF difference. Methods that use as input the digitised waveforms from the detector to estimate TOF can further improve performance. Using a deep convolutional neural networks (CNNs) to estimate TOF directly from the pair of digitised detector waveforms resulted in about 20 percent improvement [4], compared to methods like leading edge discrimination and constant fraction discrimination. Direct sampling at multiple time points can be enabled by the last generation of FPGAs [20] and can deliver more accurate TOF information. In this way the need for dedicated ASICS can be avoided.

With the current detector technology (LYSO + SiPM), 100 ps at a system level may be reachable with some reduction in detector thickness, which also reduces the scintillator cost. An important evolution are the Calcium co-doped versions of LSO leading to increased light output and shorter decay time compared to current LSO(Ce) scintillator, which will lead to faster timing resolutions [57].

The future of reconstruction less PET with 10 ps TOF [33] will require new detector technologies. It will take quite some time before this technology is developed and mature enough to be introduced in clinical systems.

#### Improving the system by better detector spatial resolution and DOI

A four-layer depth-of-interaction (DOI) detector for TOF PET has been proposed by a group in Japan [64]. They have shown that using the DOI information and using a timing correction dependent on the depth can improve time resolution. As demonstration of this principle, they reported an improvement in timing resolution (measured in combination with fast BaF_2_) from 730 to 477 ps in FWHM. Monolithic scintillators are promising for this concept as they deliver accurate DOI information.

The intrinsic limit of spatial resolution in clinical PET is determined by positron physics. While in preclinical systems the limit is mostly determined by positron range, clinical systems are dominated by the effect of acolinearity [35]. For a diameter of 60–80 cm, the best possible spatial resolution is about 2 mm due to non-collinearity of the 2 gammas. The aim of the ultimate detector should be to have an intrinsic spatial resolution well below this value, so the detector itself has a limited contribution on the system spatial resolution. Our estimates [35] are that such a detector should have a intrinsic resolution better than 1.3 mm.

A detector with very good intrinsic spatial resolution alone is not sufficient to build the ultimate PET system. First of all, this property should also be combined with sufficient stopping power and very good TOF resolution. Due to the relative thick detectors, improving the intrinsic spatial resolution alone will not result in a uniform spatial resolution over the FOV. Therefore, also DOI measurements are required. None of the current PET or proposed total body PET systems has this capability. Total body systems would benefit more from this information as there is DOI in both transverse and axial directions in TB-PET.

A pixelated detector with all required properties of TOF, good intrinsic spatial resolution and DOI is hard to realise. DOI with multiple layers has always been a challenge in this type of detectors. Reducing the pixel size will lead to better spatial resolution, but also less light output and will have a negative impact on energy and TOF resolution. Monolithic detector technologies are used now frequently in preclinical systems. They are also promising for clinical systems as they can combine accurate DOI based on the light spread function, with high intrinsic spatial resolution. Very good TOF may be the most challenging parameter for this technology as the light is spread over a larger area of photodetectors. New fast algorithms for fast and accurate TOF estimation should be developed to exploit the full potential of these detectors. Very good results have been shown for detectors in a lab setup, but have not yet been realised in full systems. The cost of monolithic detectors is comparable to pixelated detectors when built as blocks with pixel sizes (2–4 mm) used in current clinical systems. The readout and positioning in this type of detectors is however more complex and costly than pixelated detectors. This may be a major limitation for building full total body PET systems based on monolithic detectors; however, the availability of more advanced Field-Programmable Gate Arrays (FPGAs) can lead to a fast development for these systems.

Based on an initial design of a paediatric PET system with long axial FOV [40], a compact total body PET design with only 3–4 times the detector material of a current PET-CT scanner has been proposed [69]. Besides only improving the sensitivity, the aim is to also take a large step in spatial resolution (approaching the limits of clinical PET) by using high-resolution monolithic detector technology. This technology has been implemented in several preclinical systems and now seems mature enough for using it in clinical systems. Compared to preclinical systems, the detector cost can be reduced by adapting the size of the crystal and the number of SiPM pixels.

## Discussion

The first total body PET systems have included TOF in their system and were combined with CT. This seems to be a logical choice as the high sensitivity of these new PET designs will lead to PET acquisition times close to the speed of CT acquisitions; also the main driver for these systems is body imaging which is primarily done with PET-CT. Combining with MR would be suboptimal as the acquisitions of total body MR would be much slower than with PET. The total cost of the system would also be significantly higher.

The first systems have mostly been the result of an academic effort and a new company on the market (United Imaging). Only one of the three major vendors (Philips via the project at UPENN) is indirectly involved in the development of a total body system. A similar situation was seen with the clinical introduction of TOF, which was first realised at the University of Pennsylvania in the Philips Gemini TF in 2006. Within 2–3 years the other vendors also introduced TOF in their PET systems. This may be a possible scenario as the current PET detector technology of all major vendors are also suitable for building total body PET systems and the full realisation of these systems is mostly an engineering challenge (cooling, countrate and image reconstruction).

The introduction of total body PET systems is a typical example of technology push introduction: research and development in new detector technology and system design brings a new imaging system to the market. It is not yet clear what the market for this new type of systems will be, but there seems to be quite some potential for existing applications and new fields can be explored. It is hard to predict how this technology will spread, but recent examples of new technology in nuclear medicine can be instructive:

In the case of PET-CT (introduced in 2000), the combination of both systems has completely taken over the standalone PET market and also led to a significant growth of PET as an imaging modality. Besides the clinical benefits of combining molecular with anatomical imaging, one of the drivers was the higher throughput: by adding a CT to PET, lengthy transmission scans can be avoided at a moderate extra cost of adding a CT scanner to the PET system. These systems have also a clear application in oncology imaging.

PET-MRI is another multimodal system introduced around 2010, but has only been a moderate success [68]. While nearly all technical challenges (like interference between modalities and the challenge of MR-based attenuation correction) have now been solved and there is a clear benefit by dose reduction, the introduction of this system in clinical routine has been limited. The main reasons for this seem to be the significantly higher cost of the system, the limited throughput (compared to PET-CT) and the lack of a clear application for combining MR with PET. Using the MR to its full extent also requires dedicated personnel. Finally, the introduction of the PET ring inside the MR also requires a wide bore MR system and is associated with a reduced performance compared to standalone MR systems. A third example is the introduction of TOF [25, 60], which was also adopted in a short period and is now also present in nearly all new PET-CT scanners [16, 66] and the most recent PET-MR systems. The combination of improved image quality with faster scanning has lead to shorter scan time and a higher throughput, and it was introduced at a reasonable extra cost. Most centres have used TOF to speed up the acquisition and only slightly reduced the administered dose to the patient. A fourth example is the introduction of SPECT-CT: this technology was introduced quite soon after PET-CT, and there has been a slow but steady introduction in the market and now most systems will be combined SPECT-CT systems. Compared to SPECT standalone (mostly done without transmission scans), the throughput is not much higher, but the additional value of the CT scan seems to justify the significantly higher cost (700 kEuro for a SPECT-CT versus 400 kEuro for a standalone SPECT).

One can expect the first introduction of total body PET in large research centres focusing on the development of novel tracers for imaging and therapy and on the use of PET in drug development. Similar as with other expensive imaging systems (7T MRI, PET-MR, linac-MRI), institutional and government funding should allow the acquisition of these high-cost systems by a reasonable number of centres in the world. The major technical advantages of these systems (simultaneous and dynamic imaging of a large part of the body, low-dose capabilities and scanning at late time points) are the key factors catching the interest of these centres, and they can have a large impact on their research.

Increasing patient throughput while preserving image quality will be the main driver for purchasing a total body PET. The use of a total body PET system will be a trade-off between reduced scanning time and reduced tracer dose and will depend on the specific situation of each PET center (capacity of tracer synthesis etc). Also, the choice of the optimal length for the axial FOV will depend heavily on the specific demands of each PET center.

## Conclusions

Since the first concept idea of total body PET in the early 1990s, the detector technology has improved significantly with regard to energy and timing resolution. Another major change since that time has been the transition of PET from a modality mostly used for fundamental and clinical research into clinical routine (accelerated by the combination with CT). Since 2000, most PET scans are related to oncology and typically a large portion of the body is acquired in these scans. This type of scans is the one in which total body PET systems have superior performance (10–40 × higher sensitivity), so there is also a clear direct application for this technology.

The combination of available detector technology with substantial funding from NIH and a major contribution from the medical imaging industry has very recently led to the realisation of the first total body PET systems: 2 systems were built for large animal imaging and 2 systems for human applications. As predicted by extensive simulations, these systems show superior performance with regard to sensitivity and at least equal performance for the other parameters (compared to the current systems). The first clinical results of these first total body PET systems have impressed the community, and it can be expected that several institutes will add it to their research equipment. The availability of this technology in the first centres and probably within the next years in other large research centres will enable the demonstration of its benefits in clinical imaging and clinical research.

The major hurdle for spreading this technology in clinical centers is the much higher cost of such scanners. As shown in this paper, for body imaging, quite large gains (9–10 ×) can already be obtained using scanners with an axial length of 70 cm and this length is already optimal for organ-specific imaging like brain scanning. Looking at the typical set of PET scans performed in a clinical center, such a system would already enable much higher throughput and enable ultralow-dose imaging for specific populations (e.g. paediatric). For institutes starting in molecular imaging, the high sensitivity of total body PET systems may justify the high cost of this system by avoiding the need for an onsite cyclotron. Before a clinical centre can justify the much higher cost for the 1-m-40- or 2-m-long axial FOV systems, it will first require demonstration of its clinical benefits in the first pioneering institutes.

## Data Availability

The datasets used in this paper are available from the corresponding author on reasonable request.
